# Free D-amino acids produced by commensal bacteria in the colonic lumen

**DOI:** 10.1038/s41598-018-36244-z

**Published:** 2018-12-17

**Authors:** Mitsuharu Matsumoto, Akihiro Kunisawa, Takanari Hattori, Shuichi Kawana, Yusuke Kitada, Hazuki Tamada, Shinichi Kawano, Yoshihiro Hayakawa, Junko Iida, Eiichiro Fukusaki

**Affiliations:** 1 Dairy Science and Technology Institute, Kyodo Milk Industry Co. Ltd, Tokyo, Japan; 2 Shimadzu Corporation, Kyoto, Japan; 3 Osaka University Shimadzu Analytical Innovation Research Laboratory, Osaka University, Osaka, Japan; 4 Graduate School of Engineering, Osaka University, Osaka, Japan

## Abstract

D-amino acids (D-AAs) have various biological activities, such as activation of *N*-methyl-D-aspartic acid (NMDA) receptor as a co-agonist by D-Ser. Since several free D-AAs are released in the broth monocultured with bacterium and D-AAs are probably utilized for bacterial communication, we presume that intestinal microbiota releases several kinds of free D-AAs, which may be involved in the hosts’ health. However, presently, only four free D-AAs have been found in the ceacal lumen, but not in the colonic lumen. Here, we showed, by simultaneous analysis of chiral AAs using high-sensitivity liquid chromatography-tandem mass spectrometry (LC-MS/MS), that 12 free D-AAs (D-Ala, D-Arg, D-Asp, D-Gln, D-Glu, D-*allo*-Ile, D-Leu, D-Lys, D-Met, D-Phe, D-Ser, and D-Trp) are produced by intestinal microbiota and identified bacterial groups belonging to Firmicutes as the relevant bacterial candidates.

## Introduction

Low-molecular weight metabolites produced by intestinal microbiota from diet- and host-derived compounds regulate intestinal microbiota and host crosstalk^1^. A large number of low-molecular weight metabolites are produced by the intestinal microbiota^2^, a part of which is probably transported to the bloodstream from the colonic lumen^3^. Although it is well-known that short-chain fatty acids contribute to the intestinal immune system and hormone secretion via G-protein-coupled proteins^4^, the function of other metabolites remains unknown, and many metabolites are yet to be identified or detected.

Previously, D-amino acids (D-AAs) were believed to be absent in mammals; however, advances in analytical technology have led to the identification of free D-AAs in mammals, a fraction of which are biologically active. For example, D-Ser activates the *N*-methyl-D-aspartic acid (NMDA) receptor as its co-agonist^5,6^. Therefore, free D-AAs in the gut lumen may be involved in the cross-talk between gut bacteria and the host via binding to epithelial cell receptors and immune cells in the lamina propria. Since several free D-AAs are released in the broth monocultured with bacterium^7^ and D-AAs are probably utilized for bacterial communication^8,9^, we presume that intestinal microbiota releases several kinds of free D-AAs. Hoeprich was the first to suggest intestinal bacteria as a source of serum D-Ala in 1965^10^, and subsequently, several groups have demonstrated intestinal bacteria to be the source of D-Ala in the blood and urine^11,12^. However, studies targeting intestinal luminal free D-AAs are limited. Bruckner and Schieber^12^ detected eleven D-AAs in rat feces; however, their data cannot be used for understanding the gut environment-host crosstalk as bacterial rupture by ethanol extraction could be responsible for the presence of intracellular D-AAs in the samples. Currently, only one study has analyzed free D-AAs in caecal samples extracted using phosphate buffered saline (PBS) and two-dimensional high-performance liquid chromatography (HPLC) and showed that except the known D-Ala, three additional D-AAs, namely, D-Asp, D-Glu, and D-Pro, were produced by the gut microbiota^13^. However, this analysis was conducted using only three mice, and therefore, is insufficient for profiling of intestinal luminal free D-AAs. Considering that various types of free D-AAs are produced by microorganisms in fermented food^11^, four or more free D-AAs could be present in the intestinal lumen, which is inhabited by various bacteria. In this study, we prepared germ-free (GF) and colonized (Ex-GF) mice and investigated gut luminal free D-AAs by simultaneous analysis of chiral AAs using high-sensitivity liquid chromatography-tandem mass spectrometry (LC-MS/MS), which separates D- and L-AA with higher resolution and can better detect trace amounts of D-AA than that by two-dimensional HPLC^14^. We observed that 12 free D-AAs are produced by intestinal microbiota and identified the relevant bacterial candidates.

## Results

### The difference in colonic luminal free chiral amino acids between GF and Ex-GF mice

**Figure 1:**
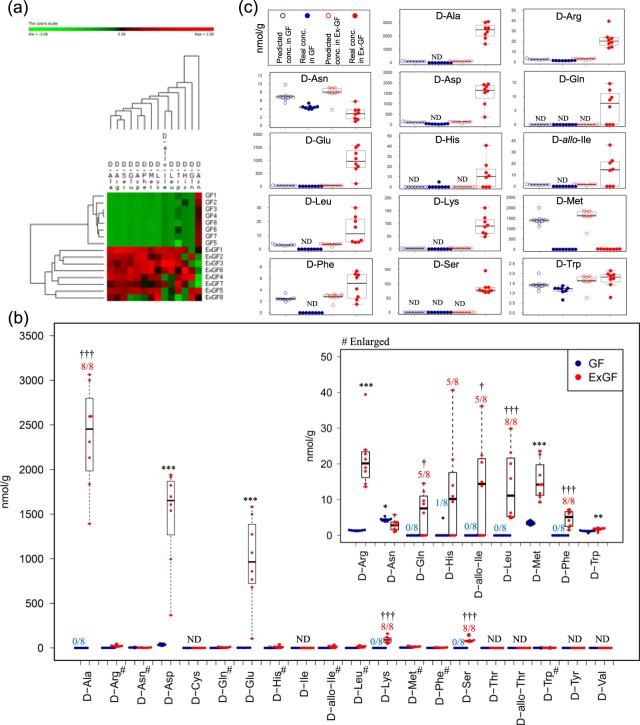
Difference in the colonic luminal D-amino acids between GF mice and Ex-GF mice. (**a**) Hierarchical clustering showing patterns of D-amino acids. Red and green indicate high and low concentrations of metabolites, respectively. (**b**) Concentrations of D-amino acids. Numbers above bars indicate incidences. *Significant difference between groups in the concentration (*p < 0.05, **p < 0.01, ***p < 0.001) by the Mann–Whitney U-test. ^†^Significant difference between groups in the incident (^†^p < 0.05, ^†††^p < 0.001) by Fisher’s extract test. ^#^These metabolites graphs are enlarged. (**c**) Quantitative comparison of D-amino acids between actual concentration in colonic content and estimated concentration when pellets reached the colon in GF mice and Ex-GF mice (nmol/g of feces). Estimated values of pellet D-amino acids in the colonic lumen were calculated as follows: estimated values of D-amino acids (nmol/g) = measured concentration in pellet/[pellet solid content (%)/solid content of colonic content (%)].

High-sensitivity LC-MS/MS chiral AA analysis detected 14 free D-AAs (D-Ala, D-Arg, D-Asn, D-Asp, D-Gln, D-Glu, D-His, D-*allo*-Ile, D-Leu, D-Lys, D-Met, D-Phe, D-Ser, and D-Trp) from the colonic content of mice. By hierarchical clustering, a remarkable difference was observed in the colonic luminal free D-AAs between GF mice and Ex-GF mice and the concentration of all D-AAs, with the exception of D-Asn in Ex-GF mice, was higher than in that in GF mice (Fig. 1a), demonstrating that intestinal microbiota significantly affects the colonic luminal D-AA (Fig. 1b). D-Ala, D-Leu, D-Lys, D-Phe, and D-Ser were detected in all Ex-GF mice, but not in GF mice (*p* < 0.001, Fisher’s extract test). Incidences of D-Gln and D-*allo*-Ile were also significantly higher in Ex-GF mice than in GF mice (*p* < 0.05, Fisher’s extract test). The concentrations of D-Arg (*p* < 0.001), D-Asp (*p* < 0.001), D-Glu (*p* < 0.001), D-Met (*p* < 0.001), and D-Trp (*p* < 0.01) were significantly higher in Ex-GF mice than in GF mice. Thus, we demonstrated that these 12 D-AAs are produced by intestinal bacteria, and this is the first report in which free D-Arg, D-Gln, D-*allo*-Ile, D-Leu, D-Lys, D-Met, D-Phe, D-Ser, and D-Trp were identified in the colonic lumen. In contrast, D-Asn concentration was lower in Ex-GF mice than in GF mice (*p* < 0.05). D-Cys, D-Ile, D-Thr, D-*allo*-Thr, D-Tyr, and D-Val were not detected in any mice. In contrast, 19 out of 20 L-AAs, with the exception of L-*allo*-Thr, were detected by this analysis. DL-Pro was not detected separately, whereas Gly was detected. Hierarchical clustering revealed a remarkable difference in the colonic luminal free L-AA content between GF mice and Ex-GF mice (Supplementary Fig. S1a). The data regarding each L-AA is shown in Supplementary Fig. S1b. The concentrations of L-Ala (*p* < 0.001), L-Arg (*p* < 0.05), L-Asp (*p* < 0.001), L-Glu (*p* < 0.001), L-His (*p* < 0.01), L-Ile (*p* < 0.01), L-Leu (*p* < 0.01), L-Met (*p* < 0.01), L-Lys (*p* < 0.01), L-Phe (*p* < 0.01), L-Ser (*p* < 0.01), L-Trp (*p* < 0.01), L-Tyr (*p* < 0.01), L-Val (*p* < 0.01), and Gly (*p* < 0.001) were significantly higher in Ex-GF mice than in GF mice. L-*allo*-Ile was abundant in Ex-GF than in GF mice (*p* < 0.01).

### Estimated value for each free chiral amino acid concentration in the colonic lumen when the pellets reached the colon

The chiral AA concentration in the pellets and the solid content of colonic matter and pellets are shown in Supplementary Table S1 and S2, respectively. We calculated the estimated value for each D-AA concentration in the colonic lumen when the pellets reached the colon. Briefly, the estimated values of D-AA (nmol/g) = measured concentration in pellet/[pellet solid content (93.5%)/solid content of colonic matter (15.9–41.5%)]. The actual and estimated concentration of D-AAs in the colonic lumen of GF mice and that of Ex-GF mice were compared (Fig. 1c), and the results confirmed that D-Gln, D-His, and D-Ser are produced by intestinal bacteria because these were not detected in the pellet, but were present in the colonic content of Ex-GF mice. Although D-Ala, D-Arg, D-Asp, D-Glu, D-*allo*-Ile, D-Leu, D-Lys, D-Met, D-Phe, and D-Val were present in the pellet, these were not detected in the colonic content of GF mice as their concentrations in GF mice colon were much lower than the estimated value, indicating that these D-AAs in the pellet are absorbed by the host in the intestinal tract. However, the concentrations of eight of these D-AAs, excluding D-Met and D-Val, in the colonic contents of Ex-GF mice were significantly higher than the estimated value, demonstrating that these D-AA are produced by intestinal bacteria. No difference was observed in the D-Trp concentration between Ex-GF mice, GF mice, and the estimated value, indicating that D-Trp is not absorbed by the host and is not affected by intestinal bacteria. Although D-Asn concentration in the colonic lumen of GF mice was lower than the estimated value, this level was not close to zero in GF mice. Moreover, no difference was detected between GF and Ex-GF mice, indicating that D-Asn absorption by the host is difficult and its level in the colon is not influenced by intestinal bacteria. These findings are summarized in Supplementary Table S3.

Almost all L-AAs, with the exception of L-Cys and L-*allo*-Thr, were detected in the pellets (Supplementary Table S1). Colonic luminal concentration of L-Asn, L-*allo*-Ile, and L-Trp in GF mice was lower than the estimated value in GF mice (Supplementary Fig. S2), indicating that these L-AAs are absorbed by the host. In contrast, the colonic concentrations of 10 L-AAs (L-Cys, L-Gln, L-His, L-Ile, L-Leu, L-Phe, L-Ser, L-Thr, L-Tyr, and L-Val) in GF mice were higher than the estimated value in GF mice, indicating that these are produced by digestive enzymes of the host from proteins contained in the pellet. The colonic concentrations of 14 L-AAs (L-Ala, L-Arg, L-Asp, L-Glu, L-His, L-Ile, L-*allo*-Ile, L-Leu, L-Met, L-Lys, L-Phe, L-Ser, L-Tyr, and L-Val)) in Ex-GF mice were higher than the estimated value in Ex-GF mice, indicating that these are produced by intestinal bacteria and are not derived from the pellet.

### The relationship between coloni**c** bacteria and free D-amino acids in Ex-GF mice

**Figure 2:**
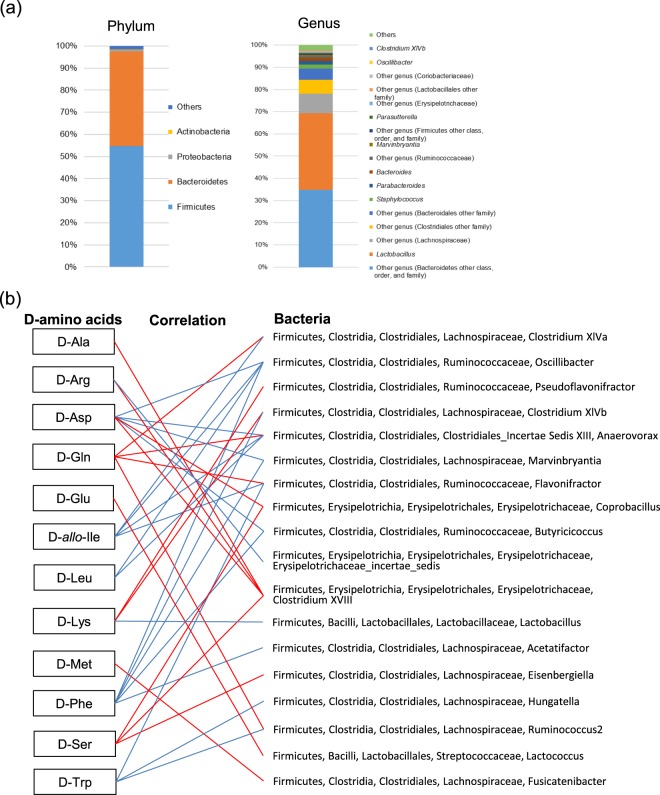
Effects of intestinal microbiota on D-amino acid production. (**a**) Relative abundances of colonic bacteria in Ex-GF mice by 16S rRNA gene amplicon sequencing. Bacteria with less than 0.1% abundance were included in others. Data of class, order, and family levels are shown in Supplementary Fig. S3. (**b**) Correlation between relative abundances of bacteria (genus level) and D-amino acid concentrations. Red and blue lines show significant positive and negative correlation, respectively. Unclassified bacteria that showed significant correlation are not shown.

Using 16S rRNA gene amplicon sequencing, we identified 7 phyla, 15 orders, 21 classes, 39 families, and 71 genera in the colonic content of Ex-GF mice (Fig. 2a, Supplementary Fig. S3). Partial least squares (PLS) regression was performed on 12 D-AAs that were produced by intestinal bacteria to estimate the colonic bacteria involved in D-AA production. As a result, the PLS regression lines of all D-AAs showed good linearity (Supplementary Fig. S4), and approximately 20 colonic bacteria having the variable influence on the projection (VIP) values of more than 1.0 were detected for each D-AA (Supplementary Table S4). Simultaneously, correlation analysis between the relative abundance of colonic bacteria and D-AA was also performed, and 1−4 key bacteria that showed significant correlation with each D-AA with high VIP value of PLS were identified (Supplementary Table S5). All bacteria, excluding unclassified bacteria, and their related D-AAs are shown in Fig. 2b. Interestingly, all listed bacteria belonging to the phylum Firmicutes; in particular, bacteria belonging to the family Lachnospiraceae showed the highest frequency, followed by bacteria belonging to the families Ruminococcaceae and Erysipelotrichaceae. All sequence data were deposited at the DDBJ Sequence Read Archive (http://trace.ddbj.nig.ac.jp/dra/) under accession number (DRA006827).

## Discussion

In this study, although we found that 12 free D-AAs (D-Ala, D-Arg, D-Asp, D-Gln, D-Glu, D-*allo*-Ile, D-Leu, D-Lys, D-Met, D-Phe, D-Ser, and D-Trp) are originated from intestinal microbiota, the origin of colonic luminal free D-AAs is unclear. Peptidoglycans of the intestinal bacterial cell wall can be one of the sources because many bacteria possess a racemase mixture of amino acids^8^, which generates free D-AAs from L-AAs and peptidoglycans containing D-AA, especially D-Ala^15^. However, whether the peptidoglycan of dead bacteria is disintegrated into a single molecule of D-AA is unclear. In contrast, several free D-AAs are released in the broth monocultured with bacterium^7^, and D-AAs affect biofilm decomposition, indicating that D-AAs are probably utilized for bacterial communication^8,9^. Therefore, we speculate that free D-AAs in the colonic lumen are released by live bacterial activity, especially those of bacteria belonging to the phylum Firmicutes. The concentrations of most L-AAs, which are the precursor of bacterial D-AAs, were higher in Ex-GF mice than in GF mice and the estimated value of pellet arrival, indicating that the intestinal bacteria produce L-AA by catabolism of protein derived from the pellet or intestinal mucus. These observations strongly suggest that intestinal bacteria produce L-AA from the pellet and mucus, and biosynthesize D-AAs from these L-AAs using a racemase mixture of amino acids.

In mammals, intrinsic D-Ser and D-Asp play important biological roles in the central nervous system and the development of endocrine function, respectively^16^; however, to the best of our knowledge, studies on the biological effect of colonic luminal free D-AAs are lacking. The study by Sasabe^13^ is the first to report the role of D-AAs in the intestinal lumen. Although this study focused on small intestinal luminal free D-AAs, it showed that D-AAs are substrates of D-amino acid oxidase (DAO), and H_2_O_2_ produced by this enzymatic reaction kills pathogenic bacteria such as *Vibrio cholerae* and *Vibrio parahaemolyticus* on the mucosal surface^13^. We speculate that free D-AAs in the colonic lumen act as agonists via binding to receptors on epithelial cells and lymphocytes in the lamina propria similarly to D-Ser in the brain. In fact, free D-Phe and D-Trp act as chemoattractants for human leukocytes through G-protein coupled receptor 109B^17^. Keeper *et al*.^18^ found that D-Trp produced by probiotics reduces secretion of chemokine ligand 17 in T-cell line, induces IL-10, and decreases LPS-induced IFN-gamma, IL-12, and IL-5 in human monocyte-derived dendritic cells. The oral supplementation of D-Trp in mice, increased the numbers of regulatory T cells in the lung and colon, decreased the lung Th2 response and ameliorated the airway allergy. However, further studies are required to unravel their specific biological functions.

D-AAs, especially D-Ser, play an essential role in NMDA receptor-mediated neurotransmission and are involved in several physiological and pathophysiological processes^16,19^. It is known that brain D-Ser is biosynthesized by serine racemase from L-Ser. However, serine racemase knockout mice display 80 to 90% decrease in D-Ser concentration in the frontal cortex, hippocampus, and striatum, indicating that 10 to 20% of D-Ser in the brain is independent on serine racemase^20^. We considered that D-Ser produced by the intestinal microbiota is a source for the brain’s D-Ser and may be transported to the brain from the intestinal lumen. Furthermore, it is well known that GF mice display increased motor activity and reduced anxiety compared with Ex-GF mice^21,22^. There is the possibility that abnormal behavior of GF mice is caused by the lack of D-Ser provided by intestinal bacteria. Further study is required to clarify the role of D-AAs with respect to behavior.

This study highlights a novel profile of intestinal luminal free D-AAs and the role of intestinal bacteria on D-AAs by high-sensitivity LC-MS/MS chiral AA analysis. Owing to its higher sensitivity, high-sensitivity LC-MS/MS can detect more types of D-AAs than two-dimensional HPLC can (which was used in a previous study^13^, in which D-Arg, D-Gln, D-*allo*-Ile, D-Leu, D-Lys, D-Met, D-Phe, D-Ser, and D-Trp were not detected). Briefly, since MS/MS can distinguish between targeted (D-AAs in this case) and untargeted chemicals, this method enables higher qualitative/quantitative analysis of D-AAs than two-dimensional HPLC does. This is suitable for the detection of trace amounts of D-AAs in the metabolome derived from natural environments such as the intestinal luminal environment. Furthermore, since the derivatization is not necessary for LC-MS/MS analysis, the time required and the pretreatment and side reactions during derivatization can be considerably reduced; furthermore, precise data can be obtained with high reproducibility. This method can be used to detect all basic chiral AAs, with the exception of Pro, for approximately 20 min per sample. In contrast, two-dimensional HPLC, which cannot analyze multiple amino acids at the same time, requires longer analysis time because it takes repeat measurements depending on the number of target amino acids. These advantages are suitable for high-throughput analysis of multiple specimens, such as fecal D-AA profiling of specific disease groups and healthy subjects. We expect that the biological role of D-AAs as low molecular weight metabolites related to intestinal indigenous bacteria-host crosstalk will be investigated using this method in the near future.

## Methods

### Mice

GF Jcl:MCH (ICR) mice were purchased from Japan Clea Inc. (Tokyo, Japan) and bred at the Research Laboratories, Kyodo Milk Industry Co. Ltd., Tokyo, Japan. Mice were housed in Trexler-type flexible film plastic isolators with sterilized tips (CLEA Japan, Inc., Tokyo, Japan) as bedding. The mice were provided with water sterilized using an autoclave (121 °C, 30 min) and commercial sterilized CMF pellets (Oriental Yeast Co. Ltd. Tokyo, Japan) *ad libitum*. Bacteriological contamination of feces was analyzed throughout the cultivation procedure using Gifu anaerobe medium (GAM) agar (Nissui, Tokyo, Japan). Six-week-old GF mice were used and divided into two groups (n = 8/group): GF mice (control) and Ex-GF mice (colonized). Using a gastric gavage tube, the stomachs of Ex-GF mice (6 weeks of age) were inoculated with 0.5 mL 1:10 diluted feces obtained from conventional ICR mice and housed for 4 weeks until specimen collection at 10 weeks. Animal experiments were approved by the Kyodo Milk Animal Use Committee (permit numbers 2017-10) and were in accordance with the Guide for the Care and Use of Laboratory Animals, published by the National Academies Press.

### Preparation of metabolome, including free D-amino acids, from colonic content and pellet

Mice were sacrificed by cervical dislocation, and the middle colonic contents were collected and frozen immediately in liquid nitrogen and stored at −80 °C until use. The colonic contents (approximately 100 mg) were diluted 10-fold with GIBCO® Dulbecco’s phosphate buffered saline (D-PBS) (Thermo Fisher Scientific, Waltham, MA, USA) and extracted twice by intense mixing for 1 min and incubating for 5 min on an icebox without agitation. One minute after the extraction, the upper aqueous portion without precipitation at the bottom was collected and centrifuged (10,000 × *g* for 10 min at 4 °C), and 100 µL supernatant was centrifugally filtered using a 5-kDa cut-off filter Ultrafree-MC (Millipore). The filtrate was stored at −80 °C until further use.

### Preparation of pellet for metabolome analysis

Sterilized commercial pellets were crushed using a mortar and prepared as described in the colonic luminal metabolome section above.

### LC-MS/MS

LC-MS/MS analysis was performed using a Nexera X2 system (Shimadzu Corporation, Kyoto, Japan) equipped with a LC-30 AD pump, a DGU-20A5R degasser, an SIL-30 AC auto sampler, a CTO-20 AC column oven, and a CBM-20A control module, coupled with an LCMS-8060 triple quadrupole mass spectrometer (Shimadzu Corporation). CROWNPAK CR-I (+) column and CROWNPAK CR-I (−) column (3 mm, internal diameter × 150 mm, width, 5 μm, Daicel Corporation) was used for the separation of D,L-AAs. The mobile phase was composed of a mixture of 80% (v/v) acetonitrile, 15% (v/v) ethanol, 5% (v/v) water, and 0.5% (v/v) trifluoroacetic acid. The flow rate, column temperature, and injection volume were set as 0.6 mL/min, 25 °C, and 1 μL, respectively. The mass spectrometer was equipped with an electrospray ionization (ESI) source under the following conditions: nebulizing gas flow, 3 L/min; heating gas flow, 5 L/min; interface temperature, 250 °C; desolvation line temperature, 250 °C; heating block temperature, 300 °C; drying gas flow, 15 L/min; interface voltage for positive mode, + 4 kV. Collision-induced dissociation gas pressure was set to 270 kPa. Data acquisition, peak selection, and integration were performed using the LabSolutions software (Shimadzu Corporation). The peak area value of each compound was normalized to that of the internal standard (DL-alanine-2,3,3,3-d4).

Previously, this method was used for analyzing chiral AAs in fecal specimens. Briefly, extracts from feces of conventional mice (n = 8) were mixed to prepare the pooled quality check samples and 35 chiral AAs were detected, and good repeatability (relative standard deviation of the peak area ratio of AAs to 13C6-L-Phe <20%) was observed in all chiral AAs (Supplementary Fig. S5). To determine the recovery of this method, 16 isotope-labeled L-AAs were added to feces prior to extraction. As a result, good recovery (80–100%) was obtained for 14 D-AAs from the feces (Supplementary Table S6).

### Measurement of the solid content of colonic matter and pellet

The solid content of colonic matter and pellet was measured using the loss on drying test. In brief, the sample was placed inside a bottle, which was dried under the same condition used for the measurement. The bottle and sample were weighed accurately and dried in a drying chamber (100 °C for 3 h). After drying completely, the drying chamber was opened, and the bottle and its content were allowed to cool to room temperature in the desiccator and weighed. The solid content was calculated from the weights of pre- and post-dried bottle and bottle with the sample.

### Extraction of bacterial DNA from colonic content

Approximately 20 mg of each sample was suspended in 600 μL of extraction buffer containing 60 mM Tris-HCl, 30 mM EDTA, and 0.8% sodium dodecyl sulfate. The suspension was mixed with 500 μL of TE-saturated phenol, incubated at 70 °C for 10 min in a water bath, and vortexed vigorously with 300 mg of glass beads (diameter, 0.1 mm) for 60 s at 4,000 rpm using a Micro Smash MS-100 homogenization system (Tomy, Tokyo, Japan). Then, 350 μL of each supernatant was collected by centrifugation (20,400 × *g*, 5 min), and the DNA was purified using Ethachinmate (Nippon Gene, Tokyo, Japan). Finally, the purified DNA was dissolved in nuclease-free water (Thermo Fisher Scientific, Waltham, MA, USA) and stored at −80 °C.

### Construction of the 16S rRNA gene amplicon library and next-generation sequencing

The V1-V2 region of the bacterial 16S rRNA gene was amplified by PCR with fusion primers using the fecal DNA as a template. The forward primer contained an Ion A adapter sequence, followed by a key, barcode, adapter (GT), and 27Fmod primer sequence (3′-AGRGTTTGATYMTGGCTCAG-5′)^23^. The reverse primer had an Ion truncated P1 adapter and 338 R primer sequence (3′-TGCTGCCTCCCGTAGGAGT-5′)^23^. PCR, DNA purification, emulsion PCR, and sequencing were performed using an Ion PGM system (Thermo Fisher Scientific), according to the manufacturer’s instructions.

### Data processing and sequence alignment

Sequence data were obtained in FASTQ format and analyzed using QIIME software^24^. Raw sequences were sorted according to the respective barcode and then screened for quality (average quality score ≥20) and primer sequence correctness. The trimmed sequences were clustered into operational taxonomic units (OTUs) at a level of 97% similarity using the UCLUST method^25^ and the farthest neighbor algorithm. The most abundant sequences in each OTU were selected as representatives. Representative sequences were aligned with the PyNAST (Python Nearest Alignment Space Termination) algorithm^26^, which is the default alignment method of QIIME, against the Greengenes core set^27^. Potentially chimeric sequences were identified and removed by the ChimeraSlayer algorithm. Non-chimeric representative sequences were assigned taxonomy using RDP classifiers with a confidence cut-off value of 80%^28^.

### Statistical analyses

Hierarchical cluster analysis (HCA) for D, L-AA quantification data was performed using the data analysis/visualization software PermutMatrix^29^. Differences in chiral AA between GF mice and Ex-GF mice were evaluated for individual metabolites using the Mann–Whitney *U*-test. The incidences of the individual chiral AAs in GF mice and Ex-GF mice were compared using the Fisher’s exact test. P-values were adjusted using the false discovery rate concept^30^, wherever essential. Partial Least Squares (PLS) analysis was performed using the multivariate analysis software SIMCA 14 (Umetrics). The results of the detected genus were used as explanatory variables, whereas the quantitative results of D-AAs were used as objective variables. Spearman’s rank correlation test was used to determine the correlation between D-AA concentrations and relative abundances of bacteria. Mann–Whitney *U*-test and Fisher’s exact test were performed using R version 3.3.2 (R Foundation for Statistical Computing, Vienna, Austria). Spearman’s rank correlation test was performed using SPSS ver.22 (IBM, Armonk, NY, USA).

## Electronic supplementary material

Supplementary Information
