# Supplementary material for: Free D-amino acids produced by commensal bacteria in the colonic lumen
Source: Sci Rep. 2018 Dec 17;8:17915. doi: 10.1038/s41598-018-36244-z (PMC6297366; doi:10.1038/s41598-018-36244-z)
Supplement: Supplementary file 1 — Supplementary Information [file 41598_2018_36244_MOESM1_ESM.pdf]

Supplementary Information

Free D-amino acids produced by commensal bacteria in the colonic lumen

Mitsuharu Matsumoto[1]*, Akihiro Kunisawa[2,3], Takanari Hattori[2,3], Shuichi Kawana[2], Yusuke Kitada[1], Hazuki Tamada[1], Shinichi Kawano[2,3], Yoshihiro Hayakawa[2], Junko Iida[2,3], Eiichiro Fukusaki[3,4]*

[1] Dairy Science and Technology Institute, Kyodo Milk Industry Co. Ltd., Tokyo, Japan
[2] Shimadzu Corporation, Kyoto, Japan
[3] Osaka University Shimadzu Analytical Innovation Research Laboratory, Osaka University, Osaka, Japan
[4] Graduate School of Engineering, Osaka University, Osaka, Japan

*Corresponding authors

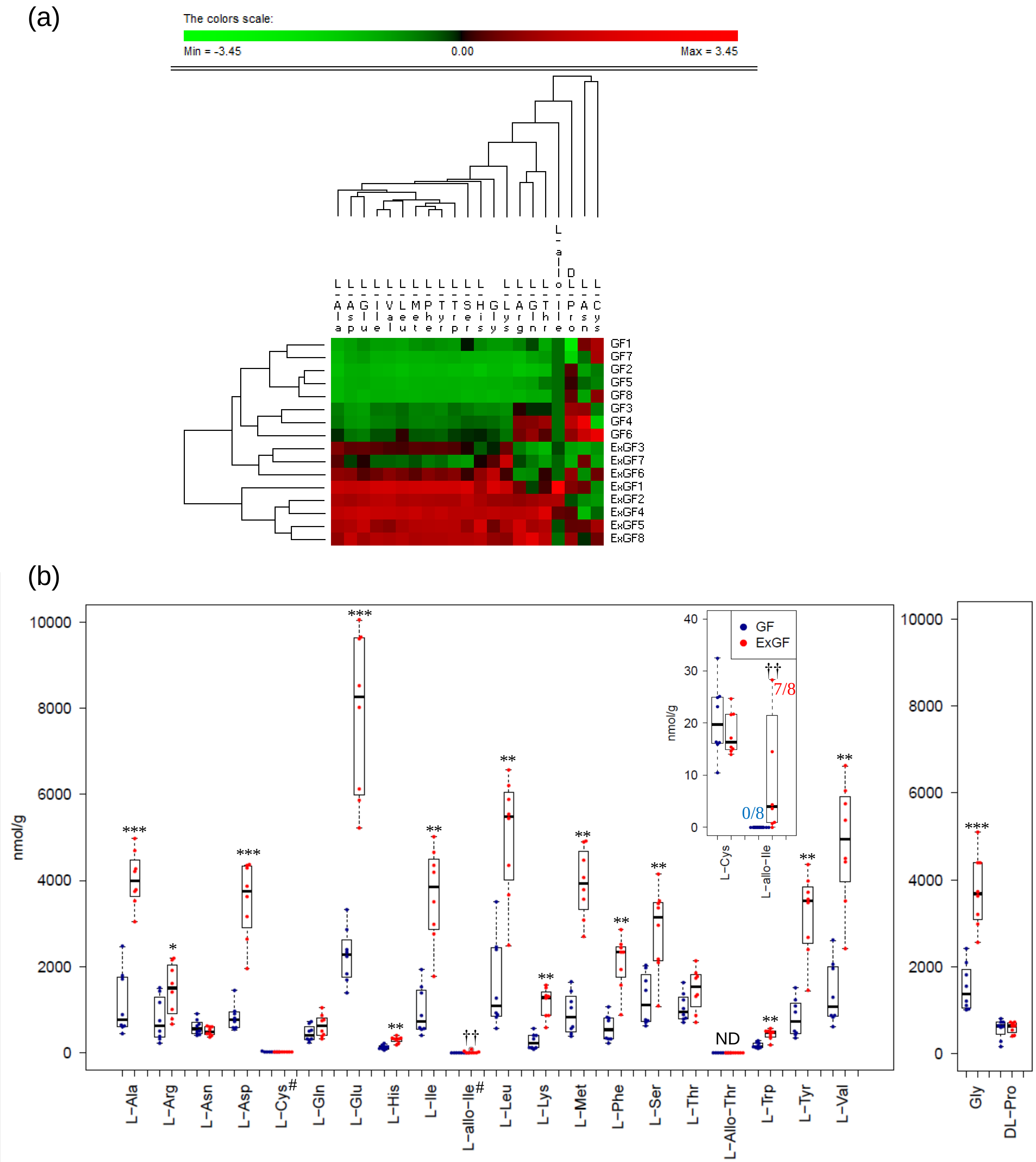

Supplementary Fig. S1. Difference in the colonic luminal L-amino acids, Gly, and DL-Pro between GF mice and Ex-GF mice. (**a**) Hierarchical clustering showing patterns of L-AAs. Red and green indicate high and low concentrations of metabolites, respectively. (**b**) Concentrations of L-AAs. Numbers above bars indicate incidences. *, Significant difference between groups in the concentration (*$p < 0.05$, **$p < 0.01$, ***$p < 0.001$) by the Mann–Whitney *U*-test. †, Significant difference between groups in the incident (††$p < 0.01$) by Fisher's extract test. #These metabolite graphs are enlarged.

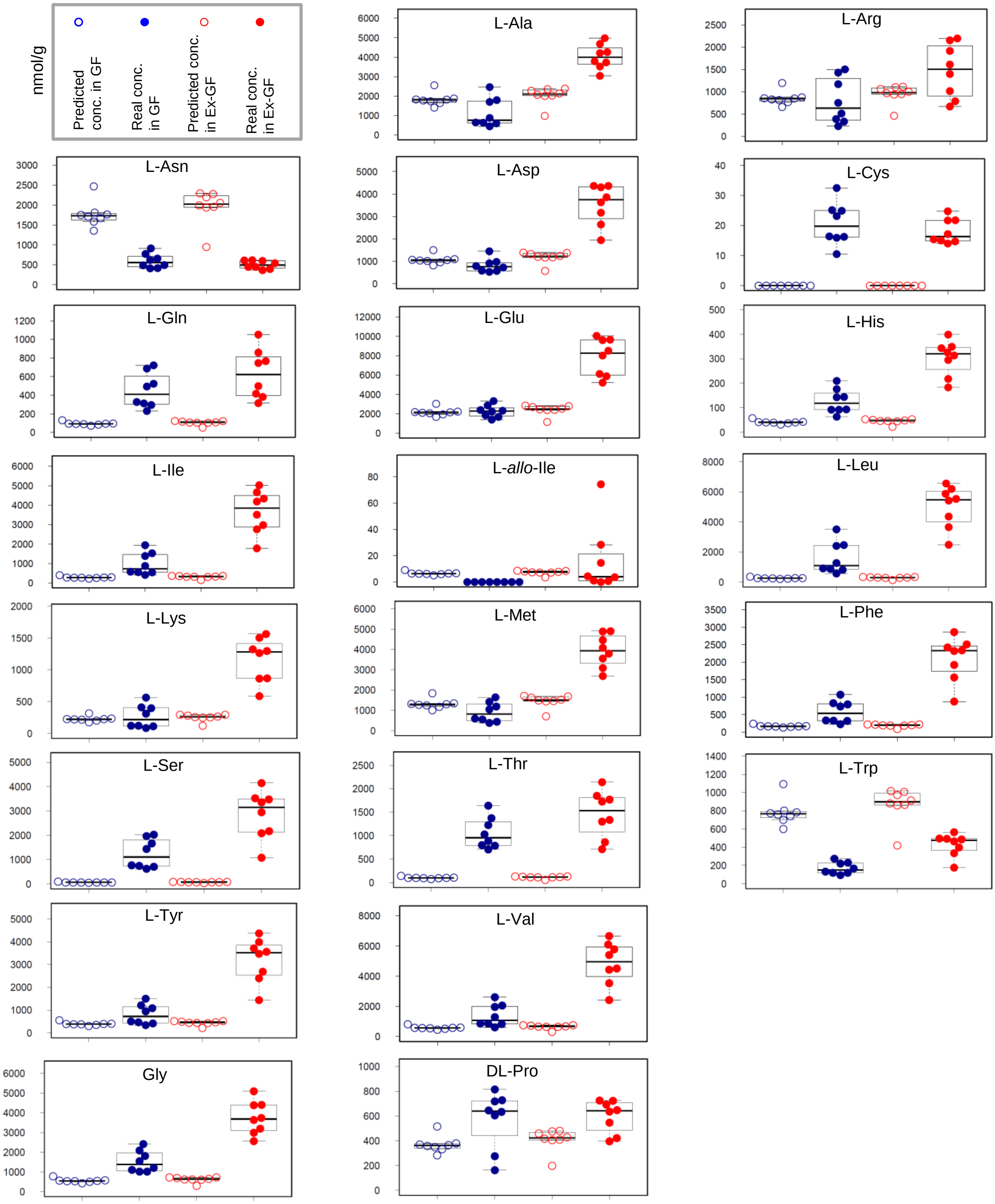

Supplementary Fig. S2. Quantitative comparison of L-amino acids between actual concentration in colonic content and estimated concentration when pellets reached the colon in GF mice and Ex-GF mice (nmol/g of feces). Estimated values of pellet L-AAs in the colonic lumen were calculated as follows: estimated values of D-amino acids (nmol/g) = measured concentration in pellet/[pellet solid content (%)/solid content of colonic content (%)]. L-*allo*-Thr was not detected from both colonic content and pellet.

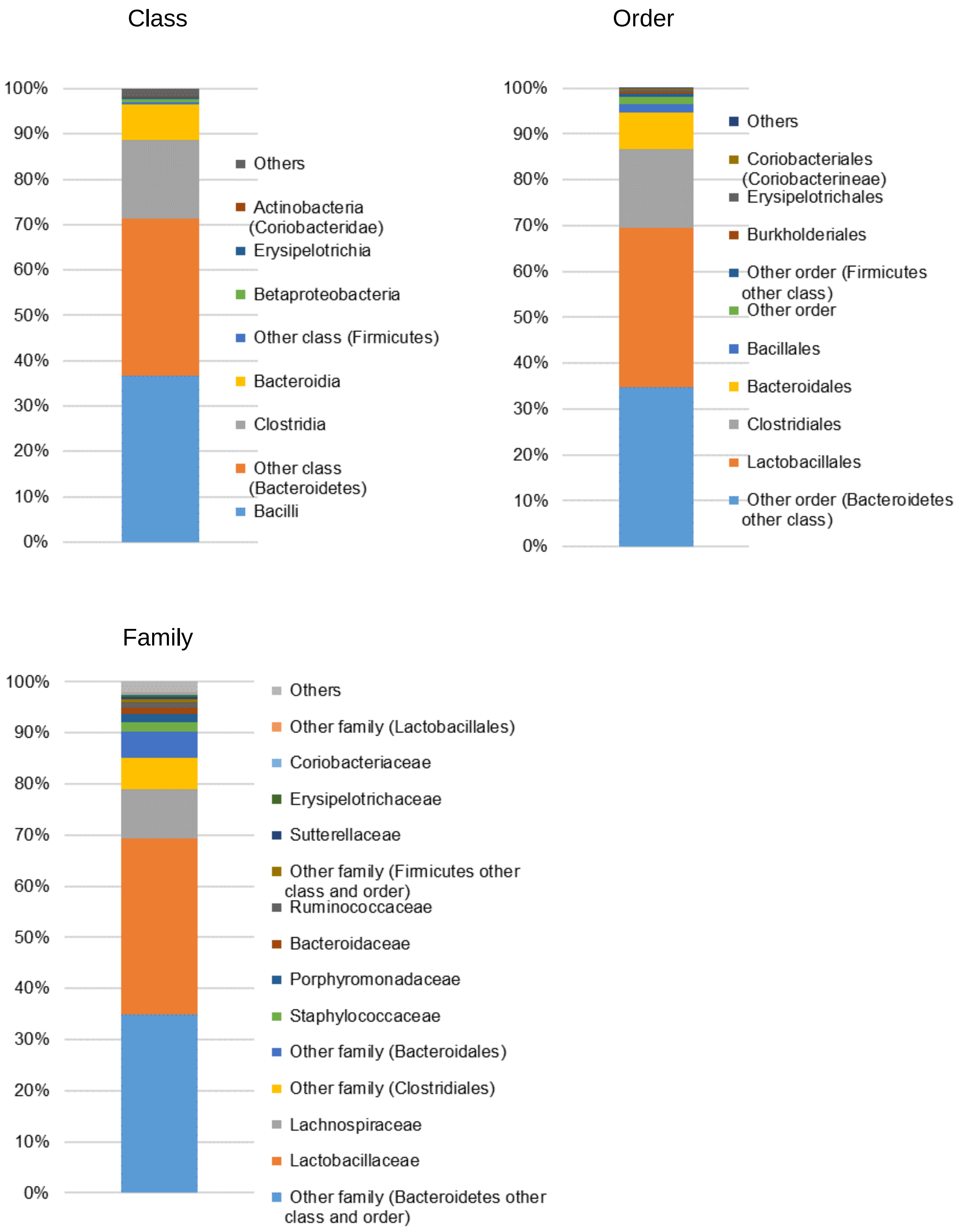

Supplementary Fig. S3. Relative abundances of colonic bacteria in Ex-GF mice by 16S rRNA gene amplicon sequencing (Class, Order, and Family). Bacteria with less than 0.1% abundance were included in others. Data of phylum and genus levels are shown in Fig. 2a.

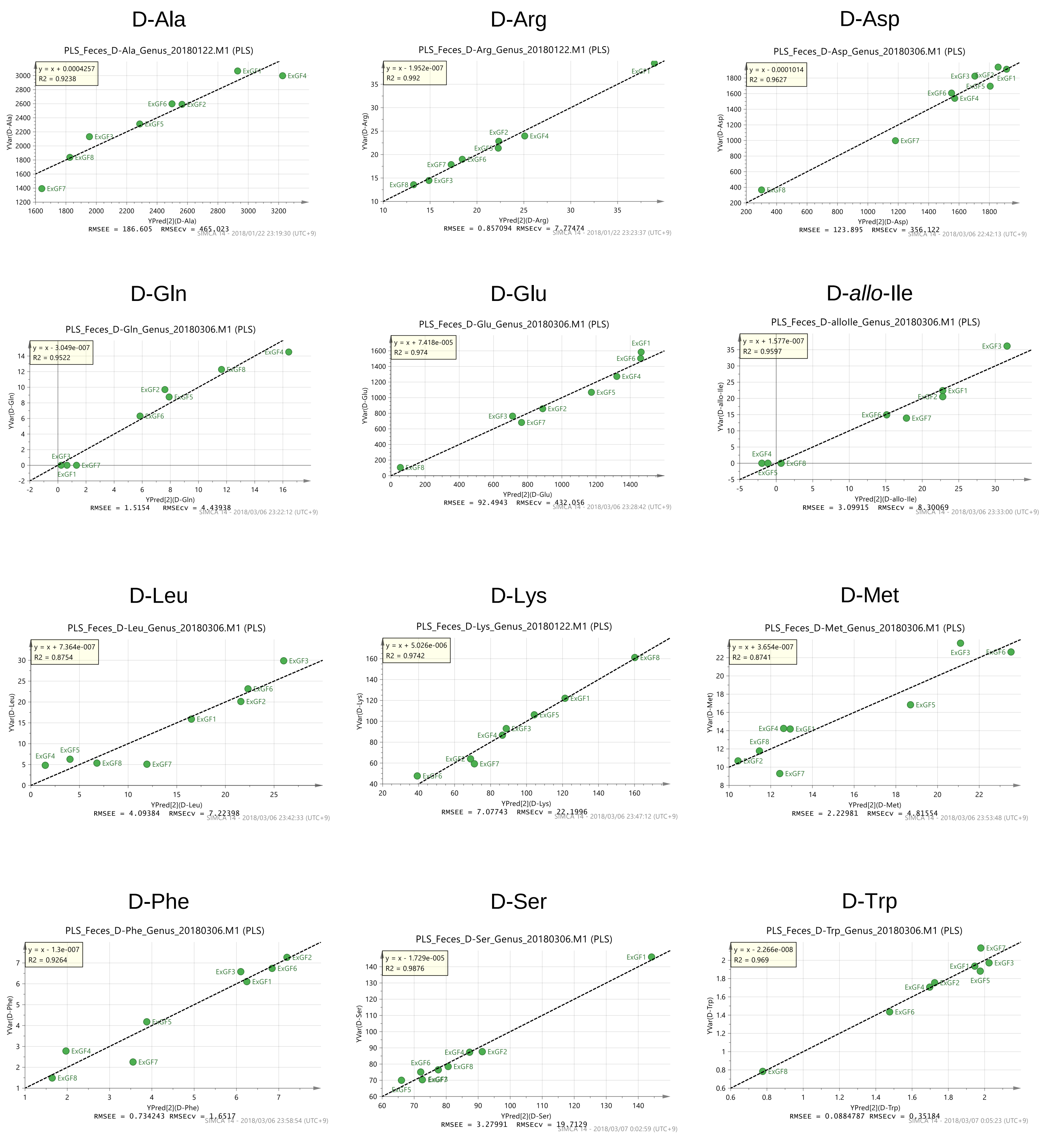

Supplementary Fig. S4. The PLS regression lines of 12 D-amino acids that were produced by intestinal bacteria

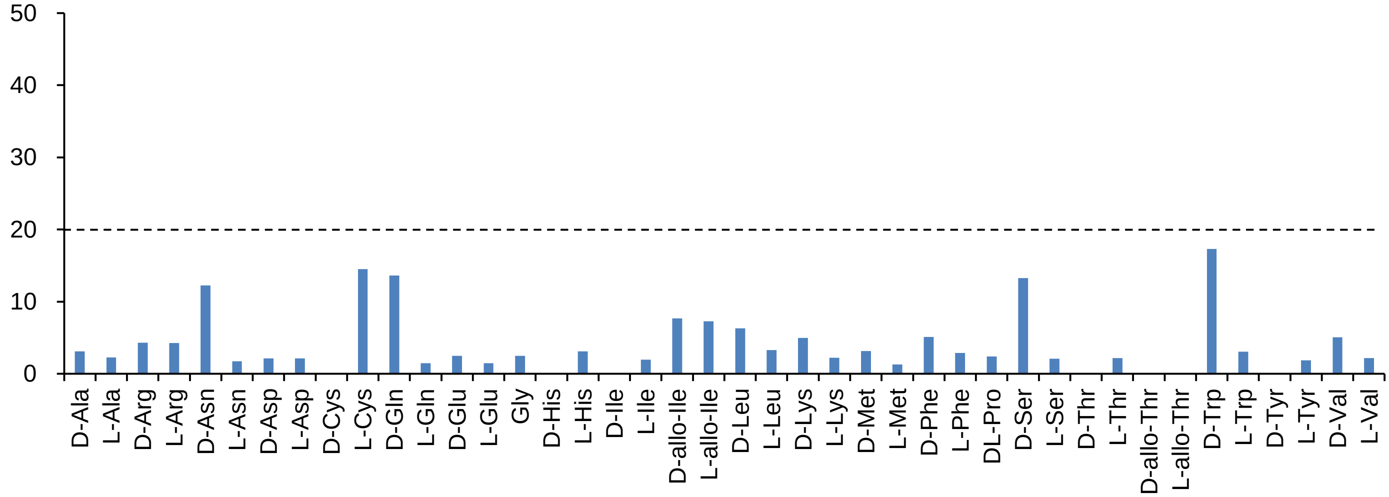

Supplementary Fig. S5. Repeatability of peak area (% Relative standard deviation)
Extract of feces from conventional mice (n = 8) were mixed to prepare the pooled quality check samples. Thirty-five chiral AAs were detected from feces, and good repeatability (RSD of the peak area ratio of AAs to 13C6-L-Phe < 20%) was observed in all chiral amino acids.

Supplementary Table S1. Concentration of D-amino acid and L-amino acids in the pellet (nmol/g)

	Mean	SD		Mean	SD
D-Ala	286.3	9.4	L-Ala	5772.3	288.6
D-Arg	8.2	0.5	L-Arg	2695.1	15.4
D-Asn	22.1	1.3	L-Asn	5555.2	53.5
D-Asp	366.5	17.8	L-Asp	3369.6	130.4
D-Cys	ND		L-Cys	ND	
D-Gln	ND		L-Gln	296.4	9.6
D-Glu	90.7	0.4	L-Glu	6849.4	42.7
D-His	ND		L-His	130.0	8.1
D-Ile	ND		L-Ile	895.1	49.8
D-*allo* -Ile	4.4	0.3	L-*allo* -Ile	20.6	1.6
D-Leu	9.4	0.3	L-Leu	832.8	53.0
D-Lys	7.2	1.1	L-Lys	716.7	49.2
D-Met	4510.3	452.4	L-Met	4138.4	401.5
D-Phe	7.8	0.2	L-Phe	540.0	21.8
D-Ser	ND		L-Ser	205.9	41.5
D-Thr	ND		L-Thr	316.8	14.7
D-*allo* -Thr	ND		L-*allo* -Thr	ND	
D-Trp	4.5	0.3	L-Trp	2466.2	139.1
D-Tyr	ND		L-Tyr	1253.8	50.1
D-Val	19.3	0.8	L-Val	1826.7	77.4
			DL-Pro	1162.8	38.2
			Gly	1747.7	64.6

ND: Not detected

Pellets were mesured in triplicates

Supplementary Table S2. Solid contents of mice colonic contents [illegible]

Mice	Solid %	Mice	Solid %
GF1	29.656	ExGF1	32.886
GF2	28.766	ExGF2	33.481
GF3	29.412	ExGF3	38.250
GF4	22.804	ExGF4	36.862
GF5	30.407	ExGF5	34.564
GF6	41.477	ExGF6	15.909
GF7	26.625	ExGF7	38.596
GF8	28.105	ExGF8	32.538
Pellet	93.451		

Supplementary Table S3. Intestinal absorption and bacterial production of each D-amino acid.

	Intestinal absorption	Bacterial production
D-Ala	+	+
D-Arg	weak	+
D-Asn	weak	-
D-Asp	+	+
D-Cys	?	-
D-Gln	?	+
D-Glu	+	+
D-His	?	weak
D-Ile	?	-
D-*allo*-Ile	+	+
D-Leu	+	+
D-Lys	+	+
D-Met	+	+
D-Phe	+	+
D-Ser	?	+
D-Thr	?	-
D-*allo*-Thr	?	-
D-Trp	-	+
D-Tyr	?	-
D-Val	+	-

+, positive; -, negative; weak, Quantities of absorption/production are low.; ?, absorption/production cannot be judged by this study.

Supplementary Table S4. Value of variable influence on projection (VIP) by PLS analysis on 12 D-amino acids that were produced by intestinal bacteria.

Bacteria	Variable Influence on the Projection											
	D-Ala	D-Arg	D-Asp	D-Gln	D-Glu	D-allo-Ile	D-Leu	D-Lys	D-Met	D-Phe	D-Ser	D-Trp
Actinobacteria, Actinobacteria (Coriobacteridae), Coriobacteriales (Coriobacterineae), Coriobacteriaceae, Enterorhabdus	1.15	1.25	1.70	1.13	1.28	1.36	1.25	1.21	1.04	1.46	1.14	1.54
Firmicutes, Other class, Other order, Other family, Other genus	1.00	1.13	1.41	1.47	1.19	1.50	1.26	1.28	1.10	1.31	1.07	1.62
Firmicutes, Clostridia, Clostridiales, Ruminococcaceae, Other genus	1.01	1.11	1.50	1.27	1.33	1.28	1.23	1.48	1.26	1.36	0.92	1.58
Firmicutes, Clostridia, Clostridiales, Clostridiales_Incertae Sedis XIII, Other genus	1.81	1.36	1.76	0.73	1.66	0.89	1.06	1.11	1.19	1.40	1.19	1.15
Firmicutes, Clostridia, Clostridiales, Lachnospiraceae, Clostridium XlVa	0.97	1.16	1.46	1.41	1.40	1.24	1.16	1.34	1.23	1.31	0.94	1.62
Firmicutes, Clostridia, Clostridiales, Ruminococcaceae, Oscillibacter	1.14	1.16	1.55	1.06	1.40	1.18	1.18	1.50	1.18	1.35	0.95	1.53
Firmicutes, Clostridia, Clostridiales, Ruminococcaceae, Pseudoflavonifractor	1.07	1.22	1.21	1.24	1.05	1.29	1.25	1.60	1.28	1.18	1.14	1.40
Firmicutes, Clostridia, Clostridiales, Lachnospiraceae, Clostridium XlVb	0.99	1.05	1.31	1.27	1.17	1.48	1.40	1.49	1.10	1.39	1.02	1.28
Firmicutes, Erysipelotrichia, Erysipelotrichales, Erysipelotrichaceae, Other genus	1.16	1.22	1.56	1.02	1.44	1.06	1.06	1.40	1.17	1.25	0.94	1.63
Bacteroidetes, Bacteroidia, Bacteroidales, Porphyromonadaceae, Parabacteroides	1.19	1.20	1.41	0.98	1.47	1.07	1.12	1.53	1.19	1.25	0.98	1.43
Firmicutes, Clostridia, Clostridiales, Clostridiales_Incertae Sedis XIII, Anaerovorax	0.59	1.06	1.19	1.76	1.01	1.66	1.53	0.98	1.15	1.48	1.33	1.06
Firmicutes, Clostridia, Clostridiales, Lachnospiraceae, Marvinbryantia	1.40	1.12	1.76	0.82	1.23	1.17	1.26	1.14	1.00	1.48	1.00	1.27
Firmicutes, Clostridia, Clostridiales, Ruminococcaceae, Flavonifractor	0.96	1.03	1.22	1.39	1.07	1.55	1.30	1.22	0.95	1.24	1.12	1.32
Firmicutes, Bacilli, Lactobacillales, Streptococcaceae, Streptococcus	1.09	1.13	1.52	0.97	1.43	0.96	0.98	1.41	1.17	1.18	0.84	1.63
Firmicutes, Clostridia, Clostridiales, Lachnospiraceae, Roseburia	1.09	1.13	1.52	0.97	1.43	0.96	0.98	1.41	1.17	1.18	0.84	1.63
Firmicutes, Clostridia, Clostridiales, Peptococcaceae 1, Peptococcus	1.09	1.13	1.52	0.97	1.43	0.96	0.98	1.41	1.17	1.18	0.84	1.63
Bacteroidetes, Bacteroidia, Bacteroidales, Other family, Other genus	1.32	1.45	1.63	0.80	1.34	0.89	0.87	1.06	1.03	1.10	1.18	1.56
Other phylum, Other class, Other order, Other family, Other genus	0.86	1.06	1.49	1.26	1.42	1.03	1.03	1.23	1.29	1.27	0.75	1.54
Firmicutes, Erysipelotrichia, Erysipelotrichales, Erysipelotrichaceae, Coprobacillus	1.14	1.75	1.06	1.04	0.76	1.23	0.84	1.29	0.58	0.99	2.22	0.80
Bacteroidetes, Bacteroidia, Bacteroidales, Bacteroidaceae, Bacteroides	1.33	1.20	1.43	0.78	1.69	0.82	0.88	1.39	1.09	1.20	0.79	1.12
Firmicutes, Bacilli, Bacillales, Planococcaceae, Sporosarcina	2.01	0.51	1.35	1.07	0.77	0.26	1.40	1.03	1.79	1.60	0.76	1.10
Firmicutes, Clostridia, Clostridiales, Ruminococcaceae, Butyricicoccus	0.67	0.92	1.36	1.36	1.11	1.14	1.11	1.19	1.18	1.38	0.71	1.40
Firmicutes, Erysipelotrichia, Erysipelotrichales, Erysipelotrichaceae, Erysipelotrichaceae_incertae_sedis	1.13	1.23	1.08	0.65	1.33	0.87	1.14	1.38	1.52	1.03	1.00	1.02
Firmicutes, Erysipelotrichia, Erysipelotrichales, Erysipelotrichaceae, Clostridium XVIII	1.57	1.47	1.38	0.71	0.83	0.85	0.88	0.82	0.86	1.43	1.63	0.67
Bacteroidetes, Bacteroidia, Bacteroidales, Rikenellaceae, Alistipes	1.53	1.31	1.10	0.70	1.44	0.83	0.91	1.12	0.89	1.12	1.27	0.85
Firmicutes, Clostridia, Clostridiales, Other family, Other genus	0.72	0.92	1.28	1.30	1.38	0.89	0.90	1.15	1.35	1.10	0.61	1.43
Firmicutes, Bacilli, Bacillales, Bacillaceae 1, Bacillus	1.31	0.93	1.08	0.67	1.04	1.23	1.36	1.01	0.79	1.23	1.17	0.87
Firmicutes, Bacilli, Lactobacillales, Lactobacillaceae, Lactobacillus	0.84	0.84	1.09	0.82	1.45	0.81	0.94	1.63	1.19	1.12	0.70	0.96
Firmicutes, Bacilli, Bacillales, Staphylococcaceae, Jeotgalicoccus	1.23	2.21	0.61	0.97	1.12	0.54	0.39	1.36	0.50	0.49	2.52	0.43
Firmicutes, Bacilli, Bacillales, Staphylococcaceae, Other genus	1.23	2.21	0.61	0.97	1.12	0.54	0.39	1.36	0.50	0.49	2.52	0.43
Firmicutes, Clostridia, Clostridiales, Ruminococcaceae, Clostridium IV	1.47	1.36	0.43	1.43	1.02	1.42	0.99	0.34	1.00	0.42	0.96	0.92
Firmicutes, Clostridia, Clostridiales, Lachnospiraceae, Acetatifactor	0.59	1.10	0.73	0.65	0.65	1.31	1.64	1.19	0.94	1.20	0.88	0.73
Firmicutes, Clostridia, Clostridiales, Ruminococcaceae, Ruminococcus	0.94	1.11	1.42	0.71	0.97	0.74	0.67	0.85	0.85	0.80	0.83	1.69
Proteobacteria, Alphaproteobacteria, Rhizobiales, Methylobacteriaceae, Methylobacterium	0.50	1.19	0.49	0.97	0.69	1.56	1.69	0.51	1.94	0.73	0.63	0.48
Firmicutes, Bacilli, Bacillales, Bacillaceae 2, Other genus	0.35	1.06	0.88	0.58	1.00	1.51	1.68	0.49	0.74	1.29	0.75	0.51
Firmicutes, Clostridia, Clostridiales, Lachnospiraceae, Eisenbergiella	1.56	1.48	0.16	0.89	0.75	0.67	0.82	1.04	0.78	0.57	1.64	0.41
Firmicutes, Clostridia, Clostridiales, Lachnospiraceae, Hungatella	0.76	0.93	0.99	0.98	0.91	0.54	0.76	0.70	0.71	0.85	0.62	1.78
Firmicutes, Bacilli, Bacillales, Other family, Other genus	0.91	1.85	0.68	0.41	0.95	0.45	0.92	1.22	0.53	0.21	1.61	0.78
Actinobacteria, Actinobacteria (Coriobacteridae), Coriobacteriales (Coriobacterineae), Coriobacteriaceae, Other genus	0.72	0.96	0.47	1.16	0.80	0.64	0.33	1.51	0.99	0.42	1.28	0.65
Firmicutes, Bacilli, Other order, Other family, Other genus	0.69	0.50	1.01	1.01	0.58	0.60	0.84	1.12	0.89	1.40	0.57	0.59
Firmicutes, Clostridia, Clostridiales, Ruminococcaceae, Intestinimonas	0.88	1.53	0.42	0.84	0.82	0.30	0.25	1.40	0.37	0.40	1.88	0.61
Firmicutes, Clostridia, Clostridiales, Lachnospiraceae, Ruminococcus2	0.63	0.47	0.84	1.47	0.58	0.82	0.72	0.54	1.24	0.77	0.40	1.23
Firmicutes, Clostridia, Other order, Other family, Other genus	1.05	0.42	0.67	1.68	0.35	1.26	1.07	0.81	0.68	0.80	0.42	0.46
Bacteroidetes, Other class, Other order, Other family, Other genus	0.56	0.68	0.64	0.91	0.64	1.34	0.75	0.80	0.53	0.61	1.17	0.89
Firmicutes, Bacilli, Lactobacillales, Other family, Other genus	0.69	0.44	0.91	0.67	0.47	1.07	1.29	0.48	0.52	1.53	0.76	0.56
Firmicutes, Clostridia, Clostridiales, Lachnospiraceae, Other genus	0.63	0.59	0.78	0.83	0.55	1.26	1.08	0.85	0.46	0.81	0.88	0.55
Deferribacteres, Deferribacteres, Deferribacterales, Deferribacteraceae, Mucispirillum	1.06	0.35	0.40	0.55	0.35	0.84	1.35	0.47	0.73	1.01	0.70	1.05
Bacteroidetes, Bacteroidia, Bacteroidales, Porphyromonadaceae, Other genus	0.66	0.58	0.78	0.68	0.55	1.12	0.95	0.83	0.50	0.77	0.91	0.54
Firmicutes, Bacilli, Lactobacillales, Streptococcaceae, Lactococcus	1.51	0.64	0.41	1.05	1.42	0.99	0.50	0.39	0.68	0.28	0.26	0.43
Proteobacteria, Gammaproteobacteria, Enterobacteriales, Enterobacteriaceae, Other genus	1.14	1.03	0.24	0.30	0.66	0.23	0.70	0.36	0.12	1.09	1.31	1.22
Proteobacteria, Alphaproteobacteria, Sphingomonadales, Erythrobacteraceae, Altererythrobacter	0.38	0.50	0.25	0.32	0.95	0.25	0.88	1.05	1.52	0.77	0.55	0.93
Proteobacteria, Deltaproteobacteria, Other order, Other family, Other genus	0.38	0.50	0.25	0.32	0.95	0.25	0.88	1.05	1.52	0.77	0.55	0.93
Verrucomicrobia, Verrucomicrobiae, Verrucomicrobiales, Verrucomicrobiaceae, Akkermansia	0.38	0.50	0.25	0.32	0.95	0.25	0.88	1.05	1.52	0.77	0.55	0.93
Firmicutes, Clostridia, Clostridiales, Eubacteriaceae, Anaerofustis	1.12	0.44	0.42	0.69	1.31	1.04	0.60	0.32	1.46	0.38	0.35	0.20
Firmicutes, Clostridia, Clostridiales, Lachnospiraceae, Lachnospiracea_incertae_sedis	1.04	0.30	0.65	1.60	0.42	1.12	0.90	0.26	0.54	0.30	0.33	0.23
Firmicutes, Clostridia, Clostridiales, Lachnospiraceae, Dorea	0.37	0.28	0.69	0.92	0.63	0.47	0.54	0.61	1.35	1.00	0.42	0.31
Firmicutes, Clostridia, Clostridiales, Lachnospiraceae, Murimonas	0.68	0.20	0.57	1.12	0.57	1.30	1.10	0.16	0.20	0.60	0.53	0.40
Firmicutes, Clostridia, Clostridiales, Catabacteriaceae, Catabacter	0.90	0.24	0.36	1.30	0.60	1.27	1.12	0.06	0.17	0.76	0.33	0.24
Firmicutes, Clostridia, Clostridiales, Lachnospiraceae, Fusicatenibacter	0.16	0.69	0.84	0.16	0.40	0.13	0.76	0.17	1.91	0.77	1.04	0.31
Actinobacteria, Actinobacteria (Coriobacteridae), Coriobacteriales (Coriobacterineae), Coriobacteriaceae, Parvibacter	0.74	0.45	0.43	0.84	0.57	0.31	0.49	0.58	1.18	0.47	0.51	0.36
Proteobacteria, Betaproteobacteria, Burkholderiales, Sutterellaceae, Parasutterella	0.16	0.47	0.15	0.49	0.63	1.06	0.80	0.88	0.98	0.08	0.30	0.35
Actinobacteria, Actinobacteria (Actinobacteridae), Bifidobacteriales, Bifidobacteriaceae, Bifidobacterium	1.00	0.27	0.12	1.21	0.51	0.90	0.86	0.12	0.25	0.84	0.07	0.08
Bacteroidetes, Bacteroidia, Bacteroidales, Porphyromonadaceae, Barnesiella	1.00	0.27	0.12	1.21	0.51	0.90	0.86	0.12	0.25	0.84	0.07	0.08
Bacteroidetes, Bacteroidia, Bacteroidales, Prevotellaceae, Prevotella	1.00	0.27	0.12	1.21	0.51	0.90	0.86	0.12	0.25	0.84	0.07	0.08
Firmicutes, Clostridia, Clostridiales, Clostridiaceae 1, Clostridium sensu stricto	1.00	0.27	0.12	1.21	0.51	0.90	0.86	0.12	0.25	0.84	0.07	0.08
Firmicutes, Clostridia, Clostridiales, Lachnospiraceae, Anaerostipes	1.00	0.27	0.12	1.21	0.51	0.90	0.86	0.12	0.25	0.84	0.07	0.08
Proteobacteria, Betaproteobacteria, Neisseriales, Neisseriaceae, Neisseria	1.00	0.27	0.12	1.21	0.51	0.90	0.86	0.12	0.25	0.84	0.07	0.08
Firmicutes, Bacilli, Lactobacillales, Enterococcaceae, Enterococcus	0.27	0.18	0.74	0.34	0.30	0.83	0.64	0.29	0.32	0.36	0.68	0.53
Firmicutes, Clostridia, Clostridiales, Lachnospiraceae, Lachnobacterium	0.27	0.18	0.74	0.34	0.30	0.83	0.64	0.29	0.32	0.36	0.68	0.53
Firmicutes, Clostridia, Clostridiales, Ruminococcaceae, Anaerotruncus	0.27	0.18	0.74	0.34	0.30	0.83	0.64	0.29	0.32	0.36	0.68	0.53
Firmicutes, Bacilli, Bacillales, Staphylococcaceae, Staphylococcus	0.36	0.61	0.20	0.45	1.11	0.50	0.16	0.06	0.81	0.27	0.36	0.06

0.50	0.60	0.70	0.80	0.90	1.00	1.10	1.20	1.30	1.40	1.50

Bacteria showing more than 0.1 % relative abundance are shown in red.

Supplementary Table S5. Correlation between D-amino acid concentration and relative abundance of colonic bacteria in Ex-GF mice.

	r-value											
	D-Ala	D-Arg	D-Asp	D-Gln	D-Glu	D-allo-Ile	D-Leu	D-Lys	D-Met	D-Phe	D-Ser	D-Trp
Actinobacteria, Actinobacteria (Coriobacteridae), Coriobacteriales (Coriobacterineae), Coriobacteriaceae, Enterorhabdus	-0.610	-0.561	-0.878	0.250	-0.512	-0.775	-0.561	0.073	-0.293	-0.756	-0.610	-0.293
Firmicutes, Other class, Other order, Other family, Other genus	-0.095	-0.143	-0.548	0.805	-0.167	-0.903	-0.500	0.238	-0.095	-0.452	-0.238	-0.762
Firmicutes, Clostridia, Clostridiales, Ruminococcaceae, Other genus	0.119	0.190	-0.381	0.732	-0.071	-0.756	-0.714	0.619	-0.333	-0.619	0.238	-0.524
Firmicutes, Clostridia, Clostridiales, Clostridiales_Incertae Sedis XIII, Other genus	-0.905	-0.738	-0.500	0.146	-0.881	-0.439	-0.238	0.119	-0.429	-0.476	-0.524	-0.048
Firmicutes, Clostridia, Clostridiales, Lachnospiraceae, Clostridium XlVa	-0.357	-0.262	-0.524	0.830	-0.548	-0.805	-0.619	0.310	-0.262	-0.571	-0.119	-0.476
Firmicutes, Clostridia, Clostridiales, Ruminococcaceae, Oscillibacter	-0.381	-0.286	-0.762	0.268	-0.333	-0.805	-0.714	0.476	-0.238	-0.905	-0.405	-0.190
Firmicutes, Clostridia, Clostridiales, Ruminococcaceae, Pseudoflavonifractor	0.071	0.167	-0.238	0.512	-0.048	-0.634	-0.548	0.738	-0.333	-0.548	0.167	-0.405
Firmicutes, Clostridia, Clostridiales, Lachnospiraceae, Clostridium XlVb	-0.167	-0.071	-0.476	0.415	-0.262	-0.683	-0.667	0.786	-0.167	-0.810	-0.048	-0.190
Firmicutes, Erysipelotrichia, Erysipelotrichales, Erysipelotrichaceae, Other genus	-0.571	-0.762	-0.595	0.220	-0.476	-0.512	0.000	0.190	0.310	-0.381	-0.690	-0.405
Bacteroidetes, Bacteroidia, Bacteroidales, Porphyromonadaceae, Parabacteroides	-0.571	-0.524	-0.429	0.366	-0.667	-0.512	-0.333	0.619	0.048	-0.619	-0.310	-0.095
Firmicutes, Clostridia, Clostridiales, Clostridiales_Incertae Sedis XIII, Anaerovorax	-0.381	-0.286	-0.714	0.781	-0.500	-0.927	-0.738	0.190	-0.310	-0.690	-0.262	-0.500
Firmicutes, Clostridia, Clostridiales, Lachnospiraceae, Marvinbryantia	-0.500	-0.476	-0.786	0.000	-0.357	-0.610	-0.476	0.238	-0.262	-0.762	-0.524	-0.167
Firmicutes, Clostridia, Clostridiales, Ruminococcaceae, Flavonifractor	-0.095	-0.143	-0.548	0.805	-0.167	-0.903	-0.500	0.238	-0.095	-0.452	-0.238	-0.762
Firmicutes, Bacilli, Lactobacillales, Streptococcaceae, Streptococcus	-0.412	-0.577	-0.577	0.423	-0.577	-0.423	-0.247	0.577	-0.247	-0.577	0.082	-0.577
Firmicutes, Clostridia, Clostridiales, Lachnospiraceae, Roseburia	-0.412	-0.577	-0.577	0.423	-0.577	-0.423	-0.247	0.577	-0.247	-0.577	0.082	-0.577
Firmicutes, Clostridia, Clostridiales, Peptococcaceae 1, Peptococcus	-0.412	-0.577	-0.577	0.423	-0.577	-0.423	-0.247	0.577	-0.247	-0.577	0.082	-0.577
Bacteroidetes, Bacteroidia, Bacteroidales, Other family, Other genus	-0.667	-0.881	-0.762	0.049	-0.571	-0.342	-0.024	-0.119	0.095	-0.405	-0.595	-0.333
Other phylum, Other class, Other order, Other family, Other genus	-0.238	-0.214	-0.405	0.561	-0.548	-0.268	-0.476	0.167	-0.524	-0.429	0.452	-0.333
Firmicutes, Erysipelotrichia, Erysipelotrichales, Erysipelotrichaceae, Coprobacillus	0.439	0.561	0.708	-0.300	0.293	0.700	0.220	0.073	-0.171	0.415	0.756	0.464
Bacteroidetes, Bacteroidia, Bacteroidales, Bacteroidaceae, Bacteroides	-0.738	-0.690	-0.524	-0.024	-0.786	-0.244	-0.286	0.548	-0.071	-0.714	-0.286	0.190
Firmicutes, Bacilli, Bacillales, Planococcaceae, Sporosarcina	-0.577	-0.247	-0.412	-0.423	-0.412	-0.085	-0.412	-0.412	-0.577	-0.412	-0.412	0.577
Firmicutes, Clostridia, Clostridiales, Ruminococcaceae, Butyricicoccus	-0.452	-0.571	-0.786	0.342	-0.548	-0.512	-0.476	0.333	0.119	-0.762	-0.238	-0.214
Firmicutes, Erysipelotrichia, Erysipelotrichales, Erysipelotrichaceae, Erysipelotrichaceae_incertae_sedis	-0.546	-0.709	-0.273	0.112	-0.600	-0.168	0.136	0.655	0.300	-0.355	-0.273	-0.164
Firmicutes, Erysipelotrichia, Erysipelotrichales, Erysipelotrichaceae, Clostridium XVIII	0.762	0.714	0.810	0.122	0.571	0.512	0.357	0.000	0.119	0.690	0.786	-0.048
Bacteroidetes, Bacteroidia, Bacteroidales, Rikenellaceae, Alistipes	-0.643	-0.571	-0.476	0.122	-0.667	-0.439	-0.333	0.667	0.000	-0.714	-0.381	0.071
Firmicutes, Clostridia, Clostridiales, Other family, Other genus	-0.286	-0.190	-0.381	0.586	-0.595	-0.342	-0.548	0.286	-0.548	-0.500	0.405	-0.262
Firmicutes, Bacilli, Bacillales, Bacillaceae 1, Bacillus	-0.627	-0.436	-0.518	0.140	-0.518	-0.671	-0.436	0.382	-0.245	-0.655	-0.627	-0.027
Firmicutes, Bacilli, Lactobacillales, Lactobacillaceae, Lactobacillus	0.190	0.310	0.214	-0.366	0.405	0.220	0.167	-0.786	-0.214	0.452	-0.167	0.190
Firmicutes, Bacilli, Bacillales, Staphylococcaceae, Jeotgalicoccus	0.577	0.577	0.412	-0.423	0.577	0.423	0.082	0.412	-0.082	0.082	0.577	0.247
Firmicutes, Bacilli, Bacillales, Staphylococcaceae, Other genus	0.577	0.577	0.412	-0.423	0.577	0.423	0.082	0.412	-0.082	0.082	0.577	0.247
Firmicutes, Clostridia, Clostridiales, Ruminococcaceae, Clostridium IV	-0.592	-0.514	-0.062	-0.639	-0.483	0.447	0.218	-0.203	0.109	-0.062	-0.343	0.733
Firmicutes, Clostridia, Clostridiales, Lachnospiraceae, Acetatifactor	-0.171	0.000	-0.366	0.125	-0.122	-0.600	-0.561	0.683	-0.244	-0.708	-0.220	-0.024
Firmicutes, Clostridia, Clostridiales, Ruminococcaceae, Ruminococcus	-0.366	-0.488	-0.634	0.350	-0.415	-0.375	-0.220	-0.293	-0.415	-0.268	-0.098	-0.586
Proteobacteria, Alphaproteobacteria, Rhizobiales, Methylobacteriaceae, Methylobacterium	-0.247	-0.412	0.247	-0.423	-0.247	0.592	0.577	0.082	0.577	0.247	-0.082	0.412
Firmicutes, Bacilli, Bacillales, Bacillaceae 2, Other genus	-0.156	-0.187	0.592	-0.192	-0.265	0.671	0.655	-0.094	0.218	0.592	0.203	0.296
Firmicutes, Clostridia, Clostridiales, Lachnospiraceae, Eisenbergiella	0.571	0.476	0.143	0.512	0.262	-0.073	-0.262	0.571	-0.024	-0.119	0.810	-0.357
Firmicutes, Clostridia, Clostridiales, Lachnospiraceae, Hungatella	-0.136	-0.409	-0.245	0.419	-0.245	-0.168	0.191	-0.027	-0.136	0.082	0.109	-0.791
Firmicutes, Bacilli, Bacillales, Other family, Other genus	0.238	0.476	-0.024	-0.073	0.262	-0.268	-0.548	0.571	-0.143	-0.500	0.095	0.333
Actinobacteria, Actinobacteria (Coriobacteridae), Coriobacteriales (Coriobacterineae), Coriobacteriaceae, Other genus	0.286	0.167	0.071	0.415	0.310	-0.171	0.143	-0.690	0.381	0.476	-0.214	-0.381
Firmicutes, Bacilli, Other order, Other family, Other genus	0.548	0.619	0.619	0.073	0.619	0.146	0.310	-0.476	0.024	0.738	0.095	-0.119
Firmicutes, Clostridia, Clostridiales, Ruminococcaceae, Intestinimonas	0.048	-0.119	-0.119	-0.342	0.167	0.049	0.143	0.452	0.000	-0.190	0.000	-0.143
Firmicutes, Clostridia, Clostridiales, Lachnospiraceae, Ruminococcus2	0.190	0.024	-0.333	0.708	-0.095	-0.317	-0.333	0.071	-0.357	-0.190	0.571	-0.762
Firmicutes, Clostridia, Other order, Other family, Other genus	0.132	0.180	-0.072	0.785	-0.084	-0.577	-0.419	0.491	0.096	-0.275	0.132	-0.383
Bacteroidetes, Other class, Other order, Other family, Other genus	0.214	0.214	0.524	-0.342	0.048	0.756	0.310	0.119	0.000	0.310	0.667	0.452
Firmicutes, Bacilli, Lactobacillales, Other family, Other genus	0.286	0.095	0.667	-0.171	0.119	0.805	0.714	-0.048	0.095	0.714	0.667	-0.024
Firmicutes, Clostridia, Clostridiales, Lachnospiraceae, Other genus	0.095	0.238	-0.238	0.610	0.000	-0.756	-0.619	0.619	-0.238	-0.524	0.024	-0.381
Deferribacteres, Deferribacteres, Deferribacterales, Deferribacteraceae, Mucispirillum	-0.515	-0.263	-0.383	-0.221	-0.371	-0.405	-0.443	0.503	-0.204	-0.707	-0.503	0.383
Bacteroidetes, Bacteroidia, Bacteroidales, Porphyromonadaceae, Other genus	-0.343	-0.312	-0.312	0.351	-0.312	-0.639	-0.234	0.592	0.047	-0.452	-0.436	-0.312
Firmicutes, Bacilli, Lactobacillales, Streptococcaceae, Lactococcus	0.698	0.558	-0.063	0.325	0.761	-0.364	-0.216	-0.178	0.457	0.063	-0.051	-0.355
Proteobacteria, Gammaproteobacteria, Enterobacteriales, Enterobacteriaceae, Other genus	0.495	0.241	0.304	-0.065	0.482	0.338	0.444	-0.114	-0.025	0.495	0.444	-0.444
Proteobacteria, Alphaproteobacteria, Sphingomonadales, Erythrobacteraceae, Altererythrobacter	0.247	-0.082	-0.082	-0.085	0.412	0.085	0.412	-0.577	0.412	0.412	-0.247	-0.412
Proteobacteria, Deltaproteobacteria, Other order, Other family, Other genus	0.247	-0.082	-0.082	-0.085	0.412	0.085	0.412	-0.577	0.412	0.412	-0.247	-0.412
Verrucomicrobia, Verrucomicrobiae, Verrucomicrobiales, Verrucomicrobiaceae, Akkermansia	0.247	-0.082	-0.082	-0.085	0.412	0.085	0.412	-0.577	0.412	0.412	-0.247	-0.412
Firmicutes, Clostridia, Clostridiales, Eubacteriaceae, Anaerofustis	0.452	0.310	-0.190	0.293	0.548	-0.512	-0.238	0.214	0.548	-0.190	-0.262	-0.333
Firmicutes, Clostridia, Clostridiales, Lachnospiraceae, Lachnospiracea_incertae_sedis	0.327	0.518	0.136	0.671	0.218	-0.503	-0.409	-0.027	0.027	0.027	0.027	-0.191
Firmicutes, Clostridia, Clostridiales, Lachnospiraceae, Dorea	0.082	0.247	0.577	0.254	-0.082	0.254	0.247	-0.247	-0.412	0.577	0.412	-0.082
Firmicutes, Clostridia, Clostridiales, Lachnospiraceae, Murimonas	0.203	0.343	-0.094	0.463	0.234	-0.639	-0.452	0.156	0.265	-0.234	-0.327	-0.094
Firmicutes, Clostridia, Clostridiales, Catabacteriaceae, Catabacter	0.296	0.405	-0.156	0.559	0.265	-0.639	-0.546	0.094	0.234	-0.265	-0.171	-0.156
Firmicutes, Clostridia, Clostridiales, Lachnospiraceae, Fusicatenibacter	-0.109	-0.245	0.191	-0.252	0.109	0.084	0.518	-0.027	0.791	0.300	-0.655	0.136
Actinobacteria, Actinobacteria (Coriobacteridae), Coriobacteriales (Coriobacterineae), Coriobacteriaceae, Parvibacter	0.095	0.286	0.095	0.195	-0.119	0.146	-0.310	-0.286	-0.405	0.024	0.500	0.214
Proteobacteria, Betaproteobacteria, Burkholderiales, Sutterellaceae, Parasutterella	0.048	0.000	0.000	-0.073	-0.143	0.268	-0.119	0.429	0.119	-0.262	0.452	0.286
Actinobacteria, Actinobacteria (Actinobacteridae), Bifidobacteriales, Bifidobacteriaceae, Bifidobacterium	0.412	0.412	-0.247	0.592	0.247	-0.423	-0.577	-0.082	0.082	-0.247	0.247	-0.247
Bacteroidetes, Bacteroidia, Bacteroidales, Porphyromonadaceae, Barnesiella	0.412	0.412	-0.247	0.592	0.247	-0.423	-0.577	-0.082	0.082	-0.247	0.247	-0.247
Bacteroidetes, Bacteroidia, Bacteroidales, Prevotellaceae, Prevotella	0.412	0.412	-0.247	0.592	0.247	-0.423	-0.577	-0.082	0.082	-0.247	0.247	-0.247
Firmicutes, Clostridia, Clostridiales, Clostridiaceae 1, Clostridium sensu stricto	0.412	0.412	-0.247	0.592	0.247	-0.423	-0.577	-0.082	0.082	-0.247	0.247	-0.247
Firmicutes, Clostridia, Clostridiales, Lachnospiraceae, Anaerostipes	0.412	0.412	-0.247	0.592	0.247	-0.423	-0.577	-0.082	0.082	-0.247	0.247	-0.247
Proteobacteria, Betaproteobacteria, Neisseriales, Neisseriaceae, Neisseria	0.412	0.412	-0.247	0.592	0.247	-0.423	-0.577	-0.082	0.082	-0.247	0.247	-0.247
Firmicutes, Bacilli, Lactobacillales, Enterococcaceae, Enterococcus	-0.082	0.082	0.082	0.085	0.082	-0.423	-0.082	0.247	0.247	-0.082	-0.577	0.082
Firmicutes, Clostridia, Clostridiales, Lachnospiraceae, Lachnobacterium	-0.082	0.082	0.082	0.085	0.082	-0.423	-0.082	0.247	0.247	-0.082	-0.577	0.082
Firmicutes, Clostridia, Clostridiales, Ruminococcaceae, Anaerotruncus	-0.082	0.082	0.082	0.085	0.082	-0.423	-0.082	0.247	0.247	-0.082	-0.577	0.082
Firmicutes, Bacilli, Bacillales, Staphylococcaceae, Staphylococcus	0.024	-0.024	-0.143	-0.390	0.333	-0.171	0.071	0.071	0.286	-0.119	-0.571	0.071

Correlation coefficients showing significant correlation with D-amino acids and high VIP value (> 1) of PLS are shown in red.

Supplementary Table S6. Recovery of isotope-labeled L-amino acids (%) by the high-sensitivity LC-MS/MS chiral AA analysis

Isotope-labeled L-AAs	Recovery rate (%)
L-Ala	98.6
L-Arg	89.5
L-Asp	80.3
L-Glu	89.9
Gly	93.2
L-His	65.3
L-Ile	97.1
L-Leu	98.2
L-Lys	77.7
L-Met	93.7
L-Phe	99.2
DL-Pro	96.9
L-Ser	91.4
L-Thr	95.3
L-Tyr	98
L-Val	100

Good recovery (80–100%) was obtained for 14 D-AAs except L-His and L-Lys from feces.
